# Represent: A community engagement roadmap to improve participant representation in cancer early detection research: An Oregon case study

**DOI:** 10.3389/fpubh.2023.1110543

**Published:** 2023-03-03

**Authors:** Jessica Currier, Ignacia Arteaga, Hannah Turner-Uaandja, Bella Starling, Nora Pashayan, Christina Jäderholm, Christopher Ponce Campuzano, Jackilen Shannon

**Affiliations:** ^1^Division of Oncological Sciences, Oregon Health and Science University, Portland, OR, United States; ^2^University of Cambridge, Cambridge, United Kingdom; ^3^Vocal, Research & Innovation, Manchester University NHS Foundation Trust, in Partnership With University of Manchester, Manchester, United Kingdom; ^4^Department of Applied Health Research, University College London, London, United Kingdom; ^5^Oregon Health & Science University-Portland State University School of Public Health, Portland, OR, United States; ^6^School of Medicine, Oregon Health and Science University, Portland, OR, United States

**Keywords:** cancer early detection, research participation, underserved and unserved populations, community engagement, cancer early detection research

## Abstract

**Introduction:**

While authentic and sustained community involvement in the research process is critically important to making new technologies and interventions effective and socially acceptable, there is uneven participation across sociodemographic, racial, and ethnic communities in many research areas, including cancer early detection research. Currently, 18% of cancer in the United States impacts Hispanics and Latinos, this population accounts for < 10% of research participants. Understanding barriers and facilitators to cancer early detection research is imperative to the ultimate success of this research. Therefore, the objectives of this study were to: understand Hispanic and Latino community perspectives in participation in cancer early detection research; and identify sustainable and mutually beneficial approaches to community engagement and involvement.

**Methods:**

The Oregon Case Study, led by Oregon Health & Science University's Community Outreach, Research and Engagement (CORE) in partnership with colleagues at Vocal, a partnership between Manchester University NHS Foundation Trust and the University of Manchester and Cambridge University, adopted a participatory research approach to better understand participation in cancer early detection research from the perspectives of Oregon's Hispanic and Latino community members. We implemented two evidence-based community engagement models, the Community Engagement Studio and the Community Readiness Assessment Model. Using a facilitated format prescribed by each community engagement model, community members helped us to answer two research questions: (1) What methods help us increase participation of underrepresented communities in cancer early detection research?; and (2) How can we build trust between researchers and underrepresented communities within cancer early detection research? Quantitative (i.e., descriptive statistic) and qualitative (i.e., thematic analysis) analytic methods were used to measure and assess community knowledge, leadership, beliefs, and resources regarding participation in cancer early detection research.

**Results:**

A total of 36 Hispanic and Latino community members participated in the two community engagement models. We identified three emergent themes pertaining to participation in cancer early detection research that include: low-level awareness of cancer early detection research and opportunities for research participation, structural barriers to research participation, and uncertainty of the benefits of research participation.

**Conclusion:**

Our approach, using two evidence-based community engagement models, yielded valuable insights about perceptions of research participation for Hispanic and Latino community members. These findings, synthesized into three key themes, led to actionable recommendations to increase research participation.

## Background

While it has often been said that cancer is a non-discriminate killer, the reality is that in Oregon and the country as a whole, individuals from geographic and racial/ethnically underrepresented groups are disproportionally affected by certain types of cancer (1). Cancer is the leading cause of death among Hispanic or Latino people in the USA, accounting for 20% of deaths (2). In 2021, there were an estimated 176,600 new cancer cases and 46,500 cancer deaths among Hispanic & Latino people in the United States (3).

The best approach to reducing cancer mortality for all population groups is through effective strategies for cancer prevention and control. New technologies that are developed through cancer early detection research are one of the most promising approaches to reducing the cancer burden and saving lives (4). However, for these technologies to reduce cancer deaths in a significant and meaningful way they must work for everyone.

Participation from people from all racial and ethnic groups is crucial in translational clinical research, biorepositories, observational studies, and clinical trials (5). Suboptimal participation rates among populations that have been historically underrepresented in cancer clinical trials, including Hispanic and Latino people, are a major obstacle to the effectiveness of cancer early detection technologies developed through research (6, 7). Between 2019 and 2021, while the number of people identifying as Not Hispanic who participated in National Institutes of Health funded research rose from 87.3 to 91.1%, the percentage of participants identifying as Hispanic fell from 9.7% in 2019 to 9.3% in 2021 (8). Stated another way, Hispanic and Latinos represented 18% (60.6 million) of the U.S. population in 2019, but make up < 10% of participants in federal cancer and drug studies (3, 8, 9). The historical and current underrepresentation of minority participants in clinical trials could reduce cancer early detection and treatment effectiveness. Without adequate representation in cancer clinical trials, researchers are less likely to develop approaches or new early detection technologies that are acceptable to and work best for minority populations, including the Hispanic and Latino population, the largest ethnic minority population in Oregon (10).

### Goals of this investigation

The objectives of this case study were to: (1) understand Hispanic and Latino community perspectives in participation in cancer early detection research; and (2) to identify sustainable and mutually beneficial approaches to community engagement and involvement. This project was guided by two questions: (1) How can all communities be included in cancer early detection research?; and (2) How can trust be built between cancer early detection researchers and communities?

## Methods

This study was a collaboration among the University of Cambridge, Vocal, a partnership between Manchester University NHS Foundation Trust and the University of Manchester, the University College London, and Oregon Health & Science University Knight Cancer Institute in Oregon, U.S.A. The multidisciplinary research team brought together expertise from social anthropology, community health, epidemiology, public health, and community engagement/public and patient involvement and engagement (PPIE).

We implemented two evidence-based community engagement models the Community Engagement Studio (CES) (11–13) and the Community Readiness Assessment Model (CRAM) (14). Both models position community members as experts and active members in every step of the process. Importantly, implementation of these models were guided by principles of compensating participants for the time and expertise, meeting the community where they are, (i.e., go to the community), and being inclusive through use of the community's preferred language. Both models are described in detail below in our description of data collection activities. We elected to implement two participatory community engagement models with community representatives to develop a deep understanding of barriers and facilitators to participation in cancer early detection research among individuals in the Hispanic and Latino community (10). This approach also enabled us to identify mutually beneficial approaches to build trust and social acceptability of cancer early detection research participation by understanding individual level attitudes and community level support.

### Participants

Participants were recruited from two regions in Oregon, (i.e., Central Oregon and the Willamette Valley). Two community partners led recruitment activities using a purposive sampling approach. Potential participants were approached by two community organizations *via* email, text, and phone describing the study and inviting their participation. To be eligible, participants self-identified as Hispanic or Latino and reside in Oregon. All participants received financial compensation in acknowledgment of their contributions to this study.

### Data collection

**Figure 1:**
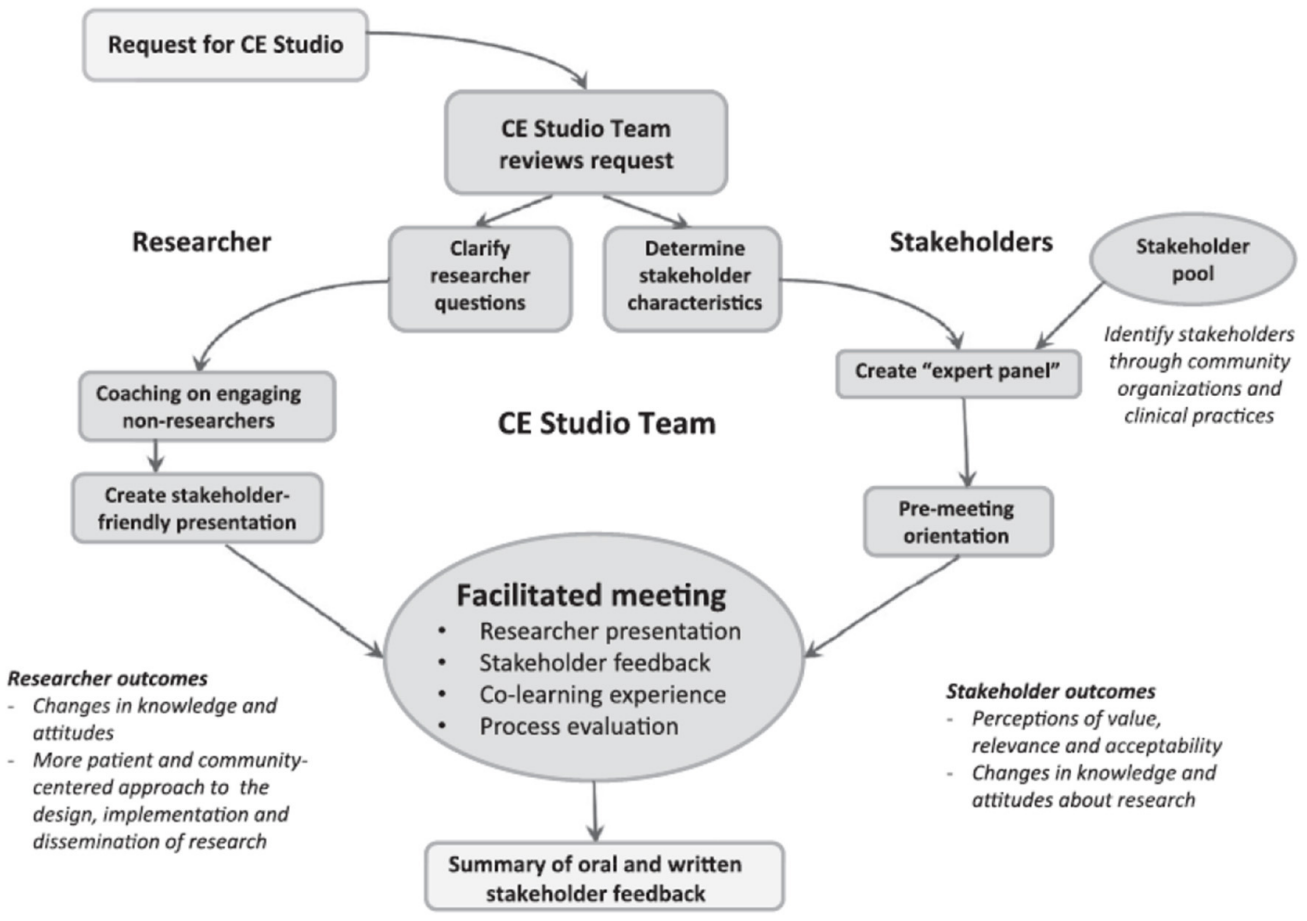
Community engagement studio framework implementation (11).

Two CES and one CRAM were implemented within a four-week period in the spring of 2022. The CES model is a facilitated conversation between identified community “experts” and the scientist (11–13). The model intentionally engages the focus population as community experts in giving them a voice to communicate with the researcher their experiential knowledge of their community, understand barriers and challenges to participating in research, identify potential ethical concerns, and provide firsthand insight into cultural and linguistic preferences (11). A structured 2-h facilitated discussion, the CES is a conversation between with community members, researcher, a facilitator, and a notetaker. The CES is an opportunity for the researcher to receive feedback from their population of interest on the relevance and feasibility of their research as shown in Figure 1.

We conducted two CES sessions within a two-week period using a virtual, web-based platform. Our partnering organization recommended the virtual format to increase participation by alleviating transportation barriers and COVID transmission concerns. Prior to the CES sessions, members of the research team met with a CES project lead with OHSU's Community Outreach, Research, and Engagement (CORE) team who provided consultation and guided them through a CES planning process, including logistical planning and the development of a 10-min presentation describing to orient CES participants to the REPRESENT project. The CES project lead also coordinated with a community partner who recruited participants and hosted both CES sessions. Both CES sessions were conducted in Spanish. Logistical planning, led by the CES project lead, involved training a bilingual OHSU CORE collaborator to facilitate the CES sessions, developing a plan to record both CES discussions, and training two bilingual notetakers who were employed by the community partner. Both sessions were video and audio recorded. Two notetakers attended each session. Notes were taken in English and given to the CES project lead who synthesized the notes in a summary report that was provided to the research team in English.

The Community Readiness Assessment model (CRAM) is a structured approach to understanding and enumerating how ready a community is to engage in different interventions, in this case, readiness to provide community wide support and buy in for cancer early detection research. Developed at the Tri-Ethnic Center at Colorado State University and revised in 2014, the CRAM involves community in every step of the process including recruitment, interviewing and analyzing the data, interpreting the results, and identifying next-steps (14). For this project, the CRAM consisted of 36 interview questions addressing the community's knowledge of efforts, leadership, community climate, knowledge of issue and resources regarding participation in cancer early detection research (Figure 1). Participation in cancer early detection research was defined as participation in ethically approved studies dedicated to understanding risk factors for developing cancer, developing new clinical tests to detect cancer early, or assessing how well current early detection tests work.

The same structured interview guide was used in all of the interviews. Bilingual, (i.e., Spanish and English) members of the Hispanic and Latino community conducted all of the interviews virtually, either by phone or a web-based platform including Zoom, and were compensated for their time. All interviewers received training *via* webinar and were sent handouts by email. A PhD researcher provided support *via* phone (call and text) and email throughout the data collection time period.

The interviews ranged from 30 min to 1-h. The interview guide included both open ended questions, as well as Likert-scale and quantitative (i.e., number between 1 and 10) assessments. Participants were given the option to conduct the interview in Spanish or English. All interviews were audio recorded, transcribed verbatim, and translated from Spanish to English by a certified translation service prior to scoring and qualitative analysis. Scoring of the interviews occurred in person with all members of the research team, our community partner, and all community members who conducted the interviews. Everybody involved in the scoring process underwent a 30-min, OHSU-developed training, including community research ethics. All community members who were a part of the interview process were compensated for their time.

### Data analysis

CES data were analyzed using thematic analysis, a qualitative descriptive approach to identifying, analyze, and report patterns in the data (15). Common themes were identified across the four sets of CES notes, (e.g., two note takers documented each CES session).

Quantitative and qualitative methods were used to analyze CRAM data. Quantitative analysis using an anchored rating scale system from the CRAM community readiness handbook was used to score the CRAM interviews. Four researchers and four community members came together in a physical space to analyze the 12 interviews. The process lasted a full day, including introductions, informal conversations, training, analysis, lunch, and reflection.

Each content area, (i.e., knowledge of efforts, leadership, community climate, knowledge of issue and resources) of the interview was analyzed and scored separately. The final outcome from the assessment is a “score” for each of these five areas, as well as a combined score. The combined score connecting all five areas informs about the overall level of engagement or community capacity and guide recommendations for “next steps”, whereas each area's score informs where to start.

All CRAM interview questions were first “scored” independently by one researcher and one community member. The two scorers then sat together to compare scores across each area for each interview they went through. If scores differed with only one point, then the average was recorded as the final score. If score discrepancies where larger than 1 “point”, then they negotiated consensus by reassessing they answers and reflecting on bias and interpretation. In the majority of cases, scorers reported having identical scores or being within 1 point from each other. This process ensured validity, but also fidelity as potential cultural differences in the interpretation of the transcript were discussed and settled.

**Figure 2:**
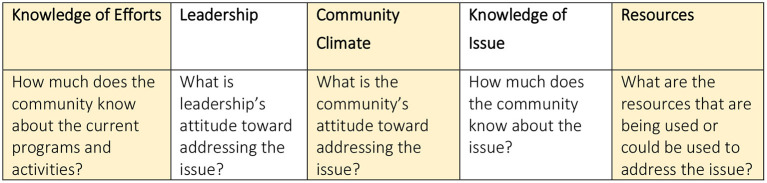
CRAM participation in cancer early detection research assessment content areas.

**Figure 3:**
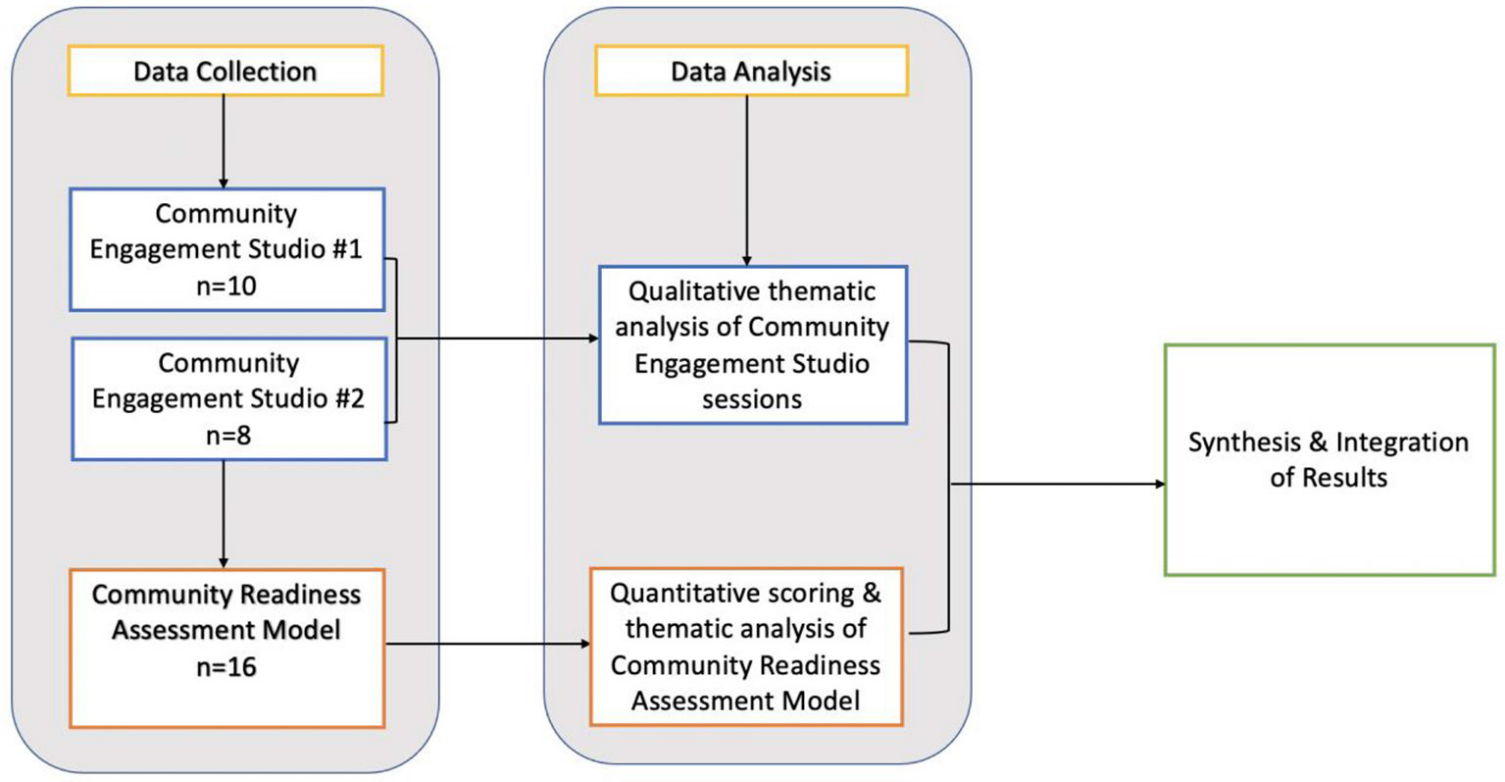
Data collection and analysis process.

Qualitative analysis was initiated in parallel with the quantitative scoring of the CRAM interviews, and completed by two coders consisting of a researcher and an intern at a later date. During the CRAM scoring, all participants had the interview transcripts as paper copies. As they read through the transcripts and assessed a score, they also highlighted passages and quotes that felt important or justified a score. The highlighted quotes were collected into a spreadsheet. A research intern also read through the interviews and extracted mentions of places/organizations, as well as specific barriers and opportunities which had not been highlighted as part of the quantitative analysis using a thematic analysis approach. Each theme aligned with the dimensions of the CRAM, (i.e., knowledge and efforts, leadership, community climate, knowledge of issue, and resources) (Figure 2). We relied on the CRAM handbook's suggested Strength, Weaknesses, Opportunities, and Threads (SWOT). Framework (16) to identify strength, weaknesses, opportunities, and threads within each theme to report results to the community in a format which aligns closely with the overall CRAM method. Our approach to data collection and analysis is shown in Figure 3.

## Results

We conducted two CES sessions and one CRAM over a 4-week period in the spring of 2022. A total of 34 individuals participated in the two community-engagement models, with 18 participating in the two CES sessions and 16 were involved in the CRAM. Participants characteristics are shown in Table 1.

**Table 1:** Participant characteristics.

**Characteristic**	**CES**	**CRAM**
Number of participants	18	16
Hispanic or Latino	18 (100%)	16 (100%)
**Age**		
Persons 25–40 years	4 (22%)	–
Persons 40–65 years	10 (55%)	–
Persons > 65 years	2 (11%)	–
Missing Data	2 (11%)	–
**Gender**		
Female	11 (61%)	10 (63%)
Male	7 (39%)	6 (37%)
Number of interviews	–	12
Number of interviewers	–	4

### CES and CRAM qualitative results

We synthesized CES and CRAM results and identified three key themes pertaining to participation in cancer early detection research. They include: (1) low-level awareness of cancer early detection research and research participation opportunities; (2) structural barriers to research participation; and (3) uncertainty of the benefits of research participation.

### Low levels of awareness of cancer early detection research

We found a general lack of understanding of the meaning of the term cancer early detection and low levels of awareness about cancer early detection research. None of the participants identified prior or existing efforts for the Latino community to participate in cancer early detection research.

- “Because I think that in general, the Latino community is not aware of the available studies. If they are not aware of the available studies, then they aren't interested either, whether the Latino population is being taken into account or not.” (CRAM Respondent #11)

Participants also stated their preference for being unaware of a health condition or disease diagnosis as there was a general feeling of hopelessness regarding ways to act on such information. From the qualitative analysis of CES and CRAM interview transcripts, we deduced that the low-level awareness about cancer early detection is mostly driven by residents' competing priorities to provide for themselves and their families, coupled with struggles to access healthcare, and high out-of-pocket costs for health care services from being uninsured or underinsured.

- “My community is living to survive not to live.” (CES Respondent #1)
- “I think that the Latino community in general is more focused on meeting other needs or has priorities in other areas, like primary care, and also economic issues and how to meet their basic needs first and foremost.” (CRAM Respondent #4)

Hispanic and Latino community members were not aware of any efforts to engage their community in cancer early detection research. Importantly, none of the participants identified prior or existing efforts for the Latino community to participate in cancer early detection research. While some participants recognized that their community's participation in cancer early detection research is important, other believed that research is only for people who are already sick.

- “These issues are certainly important and they know that research of this type can bring long-term benefits. But specific knowledge of what benefits they could bring, like what treatments are going to improve life expectancy…those things are not known at the community level.” (CRAM Respondent #9)

### Structural barriers to participation

Our study also identified several structural barriers, both individual-level and system-level, to research participation. Cultural differences, including language barriers were specifically noted as a challenge. Participants reported that research conducted in a language other than their primary language was a barrier to their participation. Others reported inaccessible, complicated language used in research documents, including consent documents, as another notable factor. In addition to language barriers, CES participants shared that health is a private matter and expressed feelings of discomfort with sharing information and being a part of research about their health. System-related factors identified by participants included limited flexibility to take time off work, cost considerations, (i.e., lost wages from time off work), lack of access to health care services, and concerns related to health insurance coverage, or lack of coverage. These concerns are consistent with published literature on barriers to underrepresented population group's participation in cancer clinical trials (5, 6, 17).

We learned that cancer is a very charged term, causing fear with some who equate a cancer diagnosis with a death sentence. We found that many Hispanic and Latino community members do not seek preventative care out of fear or lack of affordable treatment options. Having limited access to healthcare likely influences this community's reluctance to participate in clinical research.

- “You might say the word “biopsy” but what I hear is that I am going to die. It will be expensive, and I'm going to die. I am going to suffer.” (CES Respondent #9)
- “Fear of knowledge […]I think a lot of times women are scared of what they might find out.” (CRAM Respondent #7)

### Uncertain benefits of participation

We found that the benefits of participation in cancer early detection research were unclear among participants in our sample. They believed that research studies they might come across are not for them and therefore self-selected to opt out, even if they were eligible. This, we understood, was due to three reasons. Firstly, participants reported that low confidence in their English language abilities. This, combined with a lack of cultural familiarity or negative experiences interacting with providers, made them feel that they would not be understood or that nobody would help them. Secondly, the burden of participation in cancer early detection research was identified as a significant barrier. Specifically, taking time off work to participate in clinical research was equated with loss of income for hourly-wage workers. Perceptions of burdens of participation did not outweigh the benefits. Third, there were concerns about confidentiality in research studies. Some participants were undocumented, and others lacked a social security number. Considering this, they expressed worry about how the personal data required for research participation could be shared across institutions signaled a potential threat and harm.

### CRAM quantitative results

A total of 12 interviews were conducted by 4 bilingual community members over a 4-week period. Out of the 12 interviews, 9 were conducted in Spanish and 3 in English. Interviews conducted and transcribed in Spanish were translated into English for analysis by a certified translation service.

The overall score average across all 5 areas for all 12 interviews was 2.39. The results were calculated by taking the mean of each area. We also assessed the range and calculated standard deviations for each of the five areas (Table 2). Following the CRAM methodology, the mean results in each content area are all rounded down.

**Table 2:** CRAM community assessment results.

	**Knowledge of efforts**	**Leadership**	**Community climate**	**Knowledge of issue**	**Resources**
Mean	1.25	2.42	3.46	2.29	2.54
Range	1–3	1–4	3–4.5	1.5–4	2–3
SD	0.58	1.18	0.50	0.78	0.50

We found limited awareness about cancer early detection research efforts among CRAM participants in our sample. An overall score of 2.39 suggests Hispanic and Latino community members residing in Oregon's Willamette Valley have limited knowledge, attitudes and resources to address participation in cancer early detection research (Table 3). This level of readiness was expressed as incomplete information about research, low prioritization because of competing demands, scarce resources to deal with the issue, and limited understanding of early detection cancer research.

**Table 3:** Stages of readiness.

**Knowledge of efforts**	**Leadership**	**Community climate**	**Knowledge of issue**	**Resources**
**1**	**2**	**3**	**2**	**2**
**Stages of readiness**				
**No awareness**	**Denial/resistance**	**Vague awareness**	**Denial/resistance**	**Denial/resistance**
Community has no knowledge about local efforts addressing Latino participation in health-related research	Leadership believe that participation in cancer early detection research may be a concern in the community, but have shown no motivation to act	Community members believe that participation in cancer early detection research may be a concern in the community, but is not seen as a priority.	Only a few community members have knowledge about cancer early detection research, and there may be misconceptions	There are limited resources available to address Latino participation in cancer early detection research.

## Discussion

The objectives of this study were to: (1) understand Hispanic and Latino community perspectives in participation in cancer early detection research; and (2) to identify sustainable and mutually beneficial approaches to community engagement and involvement. We identified three key themes: (1) a general low-level awareness of cancer early detection research and research participation opportunities; (2) structural barriers to research participation; and (3) uncertainty of the benefits of research participation.

Our results suggest that limited knowledge about how to participate in clinical research was coupled with limited effort among researchers to engage with the Hispanic and Latino community. When studies and research are not shared with the Hispanic and Latino community, members don't know about their existence, or know that they are underrepresented in clinical research. Our results revealed that Latino and Hispanic individuals in our sample were open to participating in cancer early detection research, but they needed to: (1) be informed of research opportunities; and (2) know more about what participation entails, including the benefits of their involvement.

Further, it is necessary to raise awareness and knowledge among community members more broadly regarding the uneven rates of Hispanic and Latino participation in cancer early detection research and share opportunities for research participation. This can be accomplished by identifying networks and establishing partnerships with community-based organizations that are invested in cancer research and support efforts to increase knowledge and awareness among their members. Collaborating with stakeholders and community leaders to support the effort through strategic communication is also likely to have an impact. Many organizations hold community events. When readiness levels are low, these events could present an opportunity for face-to-face delivery of information and a space to ask and have questions answered. A small presentation, or informal talk by a community leader and researcher will draw attention to this issue and is an opportunity to provide benefits unrelated to cancer early detection research to attendees. Our findings support the position that participation in cancer early detection research should be mutually beneficial, especially in the context of limited resources and barriers to healthcare. Such an approach would help to increase cancer awareness and help to dispel the belief that a cancer diagnosis is a death sentence. This approach is also an opportunity for researchers to better understand community needs and priorities as well as build relationships.

Our participants gave insight into several potential barriers to cancer early detection research participation. The most common reasons they cited were language barriers, a lack of flexibility to take time off work, and cost considerations, (i.e., lost wages from time off work). Language barriers can be easily alleviated by researchers intentionally communicating in multiple languages and in a manner that is accessible, easily understandable, and void of overly complicated terminology. The concept of surviving vs. living was identified by Hispanic and Latino community members in our sample and may be a unique obstacle to participation in cancer early detection research for this population. Work and income related barriers are a particularly significant obstacle for clinical research participation in a population where many are focused on survival and have competing priorities (18). Many people in Oregon, including some Latino and Hispanic community members, encounter challenges in accessing the health care system, making health prevention sometimes unfeasible. This may be because people are uninsured or underinsured and have significant out-of-pocket costs for health care services. Hispanic men and women continue to be the least likely to have health insurance of any major racial or ethnic group (2). Further, there is limited knowledge of where and how to access primary are and prevention health care services and a self-described lack of awareness of how to navigate a complex health system to receive follow-up care. The literature suggests Latino and Hispanic individuals are less likely to have a primary care provider or usual source for health care compared to non-Hispanic Whites, 25 vs. 17%, respectively (2). These barriers likely influence an individual's priorities and attitudes toward participating in research.

Based on the findings, we would recommend the benefits of participating in clinical research must be meaningful enough to balance the aforementioned barriers, including lost wages from missing work. To mitigate this and other participation barriers, researchers should strive to identify and remove barriers. For example, as part of the study's design, funding and planning logistics for follow-up care for those who participate in early cancer detection research is essential, especially if the study population has limited access to healthcare. Also, financial incentives for research participation help to deter the economic burden of participation by making up for a potential loss of income from time off work, transportation, or childcare costs. Financial incentives have proven to be successful in facilitating research participation (19, 20).

Our study has a few limitations. This was a pragmatic study implemented in a real-life setting. A real strength of our approach was that the community engagement activities were delivered in Spanish. This enabled the team to recruit those that might not otherwise have participated. However, the decision to promote cultural familiarity by offering two language options brought a limitation. Our community partner hosting the CES sessions were unable to include members of the research team that did not speak Spanish. Moreover, the translation of certain experiences might not have accounted for contextual nuances. We learned that translation is essential to hear from communities that usually do not participate in early detection cancer research. Yet, we must ensure that translation goes beyond the words provided on leaflets, including contextual nuance and keeping the integrity of experiences throughout the research process so that everyone can fully participate.

Community engagement and collaboration is at the heart of any successful research. Using two evidence-based community engagement approached, we identified low-levels of awareness of cancer early detection research and research participation opportunities, structural barriers to research participation, and uncertainty of the benefits of research participation. These themes are likely influential drivers of underrepresentation of Hispanic and Latino community members in cancer early detection research. The actionable recommendations we propose are aimed at meaningfully engaging Hispanic and Latino individuals in research by removing participation barriers through trusting, reciprocal relationships between researchers and community members so that research participation is mutually beneficial.

## Data availability statement

The raw data supporting the conclusions of this article will be made available by the authors, without undue reservation.

## Author contributions

Material preparation and data collection and analysis were performed by JC, IA, CJ, HT-U, and CPC. The first draft of the manuscript was written by JC. All authors have read and approved the final manuscript. All authors contributed to the study conception and design.
